# Exploring the Synergistic Effects of MoS_2_ and PVDF for Advanced Piezoelectric Sensors: A First-Principles Approach

**DOI:** 10.3390/s25072085

**Published:** 2025-03-26

**Authors:** Rui Li, Juqi Wang, Aolin Li, Quanbin Ma, Shi Feng, Bo Ran, Lingling Zhang

**Affiliations:** 1 Xinjiang Key Laboratory of Solid-State Physics and Devices, School of Physics Science and Technology, Xinjiang University, Urumqi 830046, China; wangjuqi0805@163.com (J.W.); liaolin628@xju.edu.cn (A.L.); a17863823276@163.com (Q.M.); 18996583479@163.com (S.F.); ranbo12138@163.com (B.R.); 2 School of Material Science and Engineering, Xinjiang University, Urumqi 830046, China; 3 Center for Evidence-Based and Translational Medicine, Zhongnan Hospital of Wuhan University, Wuhan 430071, China

**Keywords:** flexible electronic devices, electrospinning, MoS_2_/PVDF composite material, piezoelectric performance, VESTA

## Abstract

Flexible wearable electronic devices have found widespread applications in health monitoring and human–machine interaction. Piezoelectric sensors, capable of converting mechanical stress into electrical signals, serve as critical components in these systems. In this study, we enhanced the piezoelectric performance of PVDF-based composite materials through MoS_2_ incorporation. Experimental results demonstrated that MoS_2_ addition effectively increased the β-phase content in PVDF, achieving a maximum value of 70.0% at an optimal MoS_2_ concentration of 0.75 wt%. Density functional theory (DFT) calculations revealed that while β-phase PVDF possesses slightly higher energy than other phases, it exhibits stronger adsorption interactions and enhanced charge transfer with MoS_2_, thereby promoting β-phase formation. The fabricated MoS_2_/PVDF composite nanofiber film maintained stable voltage output under repeated mechanical stress through 2000 operational cycles. When implemented as a body-mounted sensor, the composite material demonstrated exceptional responsiveness to human motions, confirming its practical potential for wearable electronics applications.

## 1. Introduction

With advancements in emerging nanotechnology and materials science, flexible wearable electronic devices have found extensive applications across multiple domains, including health monitoring, human–computer interaction, signal sensing, and diverse technological fields [1]. In flexible electronic devices, flexible sensors serve as core components tasked with collecting and converting various types of information (such as strain, pressure, and temperature) and acting as key intermediaries between the device and the external environment [2]. Since their invention in 2006, piezoelectric nanogenerators (PENGs) have consequently attracted significant research interest [3]. Piezoelectric material serves as the core functional element in PENG systems. These materials are characterized by their ability to convert mechanical stress into electrical energy, with critical applications spanning energy harvesting, sensor technologies, and flexible electronics.

Traditional inorganic piezoelectric materials, such as zinc oxide (ZnO) [4,5], barium titanate (BTO) [6], and lead zirconate titanate (PZT) [7], possess excellent piezoelectric properties and relatively low production costs. However, the inherent bulkiness and insufficient mechanical compliance of conventional piezoelectric materials fundamentally constrain their integration compatibility with flexible electronic systems. Polyvinylidene fluoride (PVDF), as an important piezoelectric polymer, has garnered significant attention due to its superior mechanical properties, chemical stability, and ease of processing [8]. PVDF exhibits multiple phases including α, β, γ, δ, and ε phases, among which the β-phase displays strong piezoelectricity due to the parallel alignment of dipoles [9]. Nevertheless, pristine PVDF predominantly contains the α-phase, limiting its performance in practical applications. To address this issue, researchers have attempted various methods to increase the β-phase content in PVDF to enhance its piezoelectric performance, including material doping, corona polarization, mechanical stretching, and chemical etching [10].

Electrospinning enables the preparation of nanofibers from polymer solutions under a strong electric field, simultaneously imparting mechanical stretching and electrical polarization effects [11]. PVDF can be blended with traditional inorganic piezoelectric materials to prepare precursor solutions for PVDF-based electrospun fibers, such as carbon nanotubes (CNTs) [12], barium titanate (BTO) [13,14], lead zirconate titanate (PZT) [15,16], zinc oxide (ZnO) [17], MXene [18], and others. For instance, Wang et al. incorporated modified BTO into PVDF, successfully fabricating a PENG with an output voltage of up to 2.1 V and a power density of 5.4 μW [19].

Molybdenum disulfide (MoS_2_), a two-dimensional transition metal dichalcogenide, features a large interlayer spacing, high ion retention capacity, low resistivity, high electrochemical activity, and high stability [20]. Due to the opposing orientations of adjacent atomic layers, it is considered to exhibit significant piezoelectric effects. When combined with PVDF, MoS_2_ can enhance the piezoelectric performance of the composite material.

Given this, the current study aimed to explore the application of MoS_2_ in enhancing the piezoelectric performance of PVDF-based composites. By doping MoS_2_ nanomaterials into the PVDF solution, we successfully fabricated composites with excellent piezoelectric properties. Experimental results showed that the appropriate addition of MoS_2_ increased the β-phase content of PVDF, thereby enhancing the piezoelectric activity of the material. Under conditions of 3 Hz and 3 N, the output voltage of the 0.75 wt% MoS_2_/PVDF composite sensor was measured to be 2.5 V. Additionally, we evaluated the performance of MoS_2_/PVDF composites in various application scenarios, testing its flexibility, electrical performance, filtration properties, and anti-fouling characteristics.

## 2. Materials and Methods

### 2.1. Materials

PVDF powder (average molecular weight of 3.7 × 105 g mol^−1^) was provided by Shanghai 3F New Materials Co., Ltd. (Shanghai, China). Ethanol (99.0%), *N*,*N*-dimethylmethanamide (DMF, >97.0%), and acetone (99.0%) were supplied by Tianjin Tianli Chemical Reagent Co., Ltd. (Tianjin, China). Molybdenum (IV) sulfide (MoS_2_, ≥99.5%) was obtained from Aladdin Biochemical Technologies Co., Ltd. (Shanghai, China).

### 2.2. Experimental Methods

#### 2.2.1. Preparation of MoS_2_/PVDF Composite Nanofiber Film

**Figure 1:**
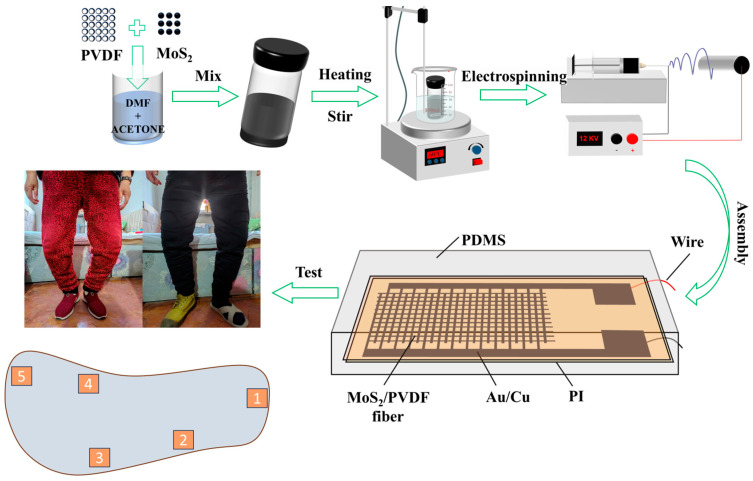
The Process of electrospinning and the fabrication of piezoelectric sensors.

DMF (6 mL) and acetone (4 mL) were placed in a beaker and mixed well. Then, MoS_2_ particles were dispersed in the mixture solvent and stirred for 30 min. The PVDF powder (1.65 g) was added to the MoS_2_ particles dispersion under continuous stirring for 3 h at 48 °C, and the dispersion was used as the electrospinning solution. The dosage of MoS_2_ particles was set at 0.25, 0.5, 0.75, and 1 wt% in different electrospinning solutions. The pure PVDF dispersion was also prepared as electrospinning solutions by the same method for comparison experiments. The MoS_2_/PVDF composite nanofiber film was fabricated by electrospinning. The electrospinning solution was loaded into a 10 mL syringe pump and electrostatically propelled through a metallic needle (inner diameter: 0.34 mm) to an electrically grounded metallic collector, maintaining a constant flow rate of 1 mL/h. The applied voltage was set at 12 kV, and the distance between the collector and the nozzle tip was fixed at 13.5 cm. All nanofibers’ films were collected on a roller with a speed of 1500 rpm for 3 h, and these experiments were performed at 30 °C under an ambient humidity of about 48% RH. The preparation of MoS_2_/PVDF composite nanofiber films is shown in Figure 1.

#### 2.2.2. Preparation of MoS_2_/PVDF Composite Sensor

MoS_2_/PVDF composite nanofiber film was used as the piezoelectric layer, an interdigital electrode was used as the electrode, and PI was used as the flexible MoS_2_/PVDF composite sensor substrate. We then cut the composite nanofiber membrane and the PI membrane (composite membrane working area of 1 × 1.5 cm^2^) and placed the interdigitated electrode on the underside of the composite nanofiber membrane as the electrode and the PI membrane as the upper cover. Then, in order to prevent contamination of the composite sensor, the composite sensor was encapsulated using PDMS. Finally, in order to avoid the triboelectric of the surrounding environment, the composite sensor was subjected to a certain pressure, and the sensor equipment was tightened by eliminating the distance between different materials. The preparation of the sensor is shown in Figure 1.

#### 2.2.3. Density Functional Theory Calculation

Density functional theory (DFT) calculations were carried out using the Vienna Ab-initio Simulation Package (VASP 5. 4. 1) [21,22]. The projector augmented wave (PAW) method and Perdew–Burke–Ernzerhof (PBE) exchange-correlation functionals were used [23,24]. The cutoff energy of the plane wave basis was set to 450 eV. We used van der Waals correction in the vdW-D3 method to properly describe the weak adsorption of PVDF on MoS_2_. All used structures were optimized with the tolerance of the Hellmann–Feynman forces set as 0.01 eV/Å.

#### 2.2.4. Characterization and Testing

The chemical composition and crystal structure of the materials were tested by Fourier transform infrared spectroscopy (VERTEX 70 RAMI, BRUKER, Berlin, Germany) and X-ray diffraction (D8 advance, BRUKER, Berlin, Germany). The morphology and surface chemical composition of the material were observed by optical microscopy, scanning electron microscopy (HITACHI-SU8600, Hitachi, Japan), and transmission electron microscopy (JEM-2100, JEOL, Tokyo, Japan). The electrical performance of piezoelectric sensors was tested by a self-assembled cyclic press, oscilloscope (OWON NDS102, OWON, Xiamen, China), and electrochemical workstation (DH7000C, Jiangsu Donghua Analytical Instrument Co., Ltd., Jingjiang, China). An electronic photo of the sample was taken with a phone.

## 3. Results and Discussion

### 3.1. Morphology and Structure of MoS_2_/PVDF Composite Nanofiber Film

**Figure 2:**
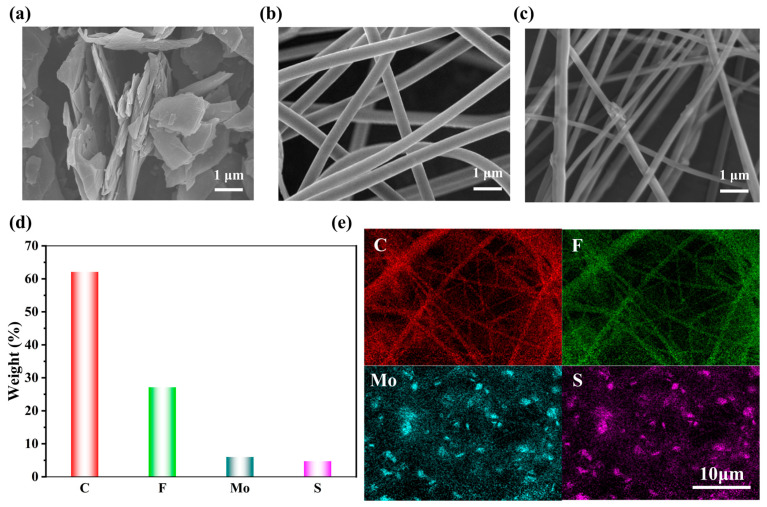
(**a**) SEM images of MoS_2_, (**b**) PVDF, (**c**) MoS_2_/PVDF composite, (**d**) elemental composition of MoS_2_/PVDF, and (**e**) mapping images.

MoS_2_ exhibits a characteristic black coloration with strong staining propensity, resulting in blackened PVDF solutions upon incorporation. Figure 2a presents the scanning electron microscopy (SEM) image revealing MoS_2_’s distinctive layered architecture. Structurally analogous to graphene in two-dimensional configuration, MoS_2_ features an S-Mo-S trilayer atomic arrangement where molybdenum atoms are covalently sandwiched between sulfur layers. Beyond structural similarities, MoS_2_ demonstrates superior electronic characteristics, particularly its exceptional in-plane electron mobility of ~100 cm^2^/(V·s)—a value surpassing that of amorphous silicon (a-Si) and comparable to advanced ultrathin semiconductors. This enhanced charge transport capability forms the principal motivation for MoS_2_ reinforcement in PVDF composites

Comparative analysis of the SEM images reveals distinct morphological differences between pure PVDF and MoS_2_/PVDF composite nanofiber mats (Figure 2b,c). While both systems exhibit smooth, continuous fibrous structures, the composite fibers demonstrate reduced diameters and enhanced alignment compared to the randomly oriented pure PVDF fibers. This organizational improvement is accompanied by increased diameter heterogeneity in the composite system, attributable to localized MoS_2_ aggregation creating thickened fiber regions alongside stretched, MoS_2_-deficient zones during electrostatic field-induced fiber elongation.

The energy-dispersive X-ray spectroscopy (EDS) maps of the MoS_2_/PVDF composite nanofiber mats are shown in Figure 2d,e. In these images, the presence of C, F, Mo, and S elements can be observed, with C and F dominating. The uniform distribution of Mo and S elements in Figure 2e indicates that MoS_2_ has been uniformly dispersed into the PVDF solution. These results demonstrate the successful preparation of MoS_2_/PVDF composite nanofiber mats.

**Figure 3:**
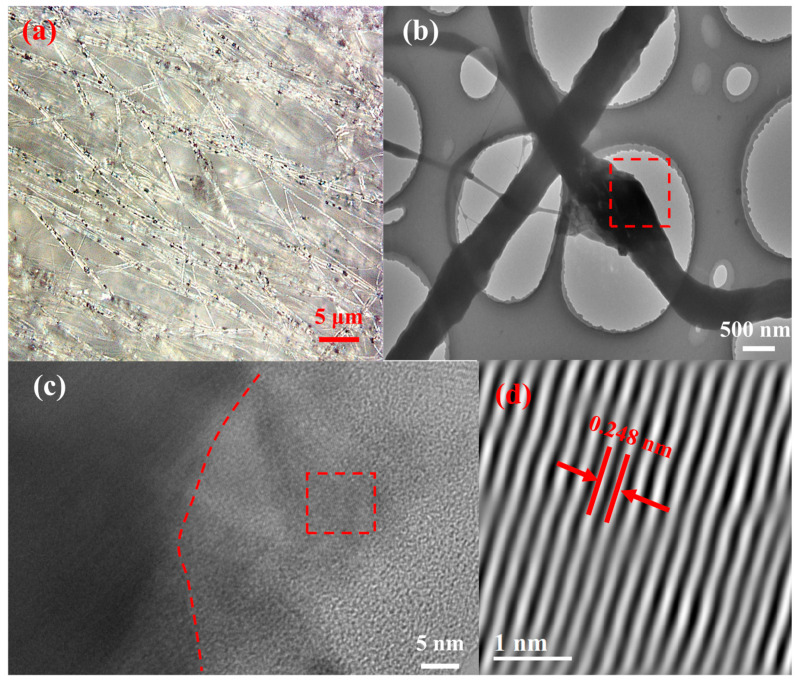
(**a**) Optical microscope image of MoS_2_/PVDF, (**b**) TEM image of MoS_2_/PVDF, (**c**) lattice image of MoS_2_/PVDF, and (**d**) lattice stripe image of MoS_2_.

Figure 3a shows the optical microscope image of the MoS_2_/PVDF composite nanofiber membrane. Due to the limited amount of molybdenum disulfide added, the black MoS_2_ appears relatively sparse in the image but is distributed quite uniformly. Observing the prepared MoS_2_/PVDF composite nanofiber membrane with TEM (Figure 3b), it can be seen that PVDF completely encapsulates the MoS_2_, and the fibers do not break at the MoS_2_ locations, indicating good dispersion in the solution and continuity during the electrospinning process. Performing lattice analysis on the red-marked area in Figure 3c reveals that the amorphous PVDF, lacking lattice fringes, surrounds the MoS_2_ with black lattice fringes. After Fourier transformation of the lattice fringes, the lattice spacing is calculated to be 0.248 nm, consistent with the (102) plane of MoS_2_.

**Figure 4:**
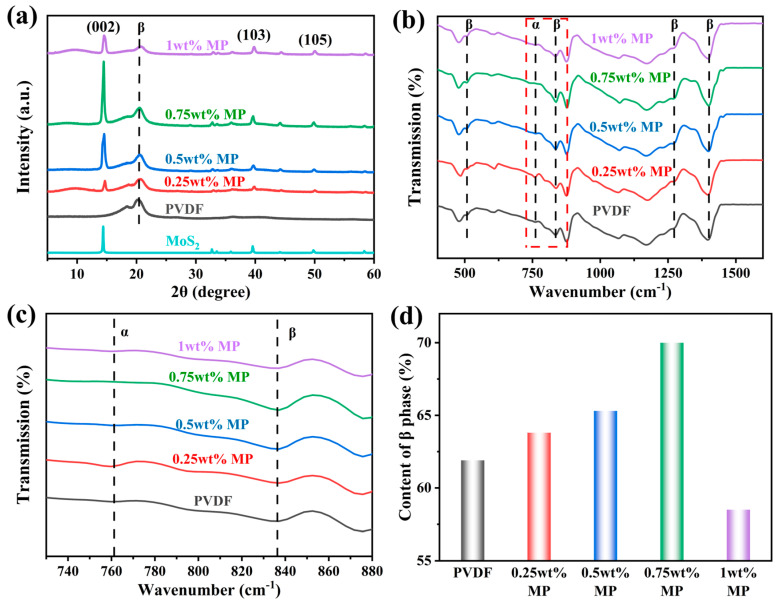
(**a**) XRD patterns, (**b**,**c**) FT-IR spectra, and (**d**) β-phase content.

The crystalline structures of PVDF-based nanofiber membranes were characterized through XRD and FTIR. The XRD spectra of pure PVDF and MoS_2_/PVDF composite nanofiber membranes with 0.25 wt%, 0.5 wt%, 0.75 wt%, and 1wt% MoS_2_/PVDF (abbreviated as: PVDF, 0.25 wt% MP, 0.5 wt% MP, 0.75 wt% MP, and 1 wt% MP) are shown in Figure 4a. PVDF possesses at least five different crystalline structures, namely the α, β, γ, δ, and ε phases [25]. The β-phase is the primary source of PVDF’s piezoelectric properties, hence, PVDF composite nanofiber membranes with high β-phase content are desirable. All five PVDF composite nanofiber membranes are dominated by the β-phase. In the XRD patterns of PVDF, the peaks at 2θ = 18.4° (020) and 20.2° (110) correspond to the non-polar α-phase and polar β-phase, respectively [26]. The β-phase crystallinity of PVDF composite nanofiber membranes increases through electrospinning and the addition of nanoparticles; however, the intensity of the β-phase decreases in the 1 wt% MP composite nanofiber membrane. This could be due to the excessive concentration of MoS_2_ making the solution too viscous, thereby hindering the effective formation of β-phase crystals during the electrospinning process. Other concentrations of MoS_2_ enhanced the β-phase content in PVDF. These results suggest that adding a certain amount of MoS_2_ can increase the β-phase content of PVDF composite nanofiber membranes. The β-phase content of PVDF nanofiber membranes was further investigated using FTIR spectroscopy. The characteristic absorption bands of the PVDF α-phase are observed at 763 cm⁻^1^, while those of the β-phase are detected at 509, 837, 1275, and 1431 cm⁻^1^, as clearly demonstrated in Figure 4b. The β-phase content of PVDF composite nanofiber membranes can be calculated using the following equation [27]:

(1) $$F(\beta )=\frac{A_{\beta }}{({Κ}_{\beta }/{Κ}_{\alpha })A_{\alpha }+A_{\beta }},$$

where A_α_ and A_β_ are the absorption peaks at 763 and 837 cm⁻^1^, corresponding to the α and β-phases of the PVDF composite nanofiber membranes (Figure 4c). K_α_ and K_β_ represent the absorption coefficients of 6.1 × 10^4^ and 7.7 × 10^4^ cm^2^ mol⁻^1^, respectively [28]. Using this equation, the F(β) values for pure PVDF and 0.25 wt%, 0.5 wt%, 0.75 wt%, and 1 wt% MP composite nanofiber membranes were calculated to be 61.9%, 63.8%, 65.3%, 70.0%, and 58.5%, respectively (Figure 4d). Among these results, the highest β-phase content was found in the 0.75 wt% MP composite. Notably, electrospinning is a viable method for achieving high β-phase content, possibly due to the stretching force, drawing, and simultaneous local polarization promoting the transformation from the α-phase to the β-phase, thereby enhancing the piezoelectric properties of the samples [29]. The β-phase represents the piezoelectric and ferroelectric characteristics of the nanofiber membranes, expected to improve the output performance of the sensor [30].

**Figure 5:**
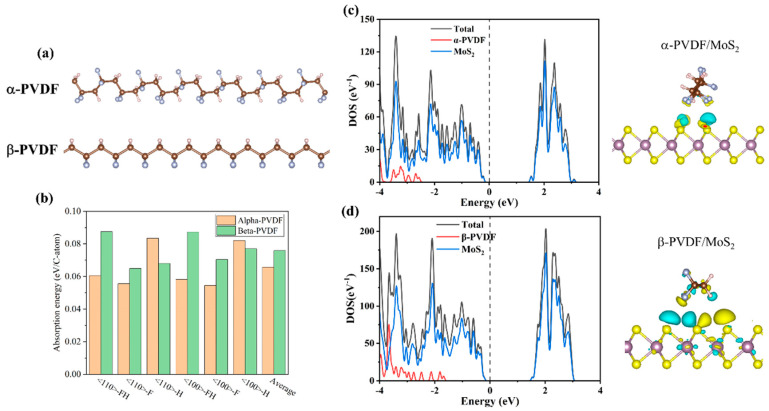
(**a**) Molecular structures of α-phase and β-phase, (**b**) adsorption energies of different configurations, density of states (DOS), and electron transfer schematics for (**c**) α-phase and (**d**) β-phase with MoS_2_.

To understand why MoS_2_ can promote the β-phase, we have performed first-principles calculations based on the density functional theory (DFT). Figure 5a presents the molecular chain configurations of the α-phase and β-phase constructed in our study. Our results show that the PVDF atomic chains in the α-phase have slightly lower energy than that of the β-phase, with an energy difference of 0.074 eV/C-atom. Such an energy difference is comparable to the adsorption energy of PVDF on MoS_2_, as displayed in Figure 5b. The adsorption energy of various MoS_2_/PVDF configurations is in the range of 0.05–0.09 eV/C-atom, indicating the interaction between PVDF and MoS_2_ is weak physical adsorption. Overall, the β-phase of PVDF exhibits relatively stronger adsorption energy with MoS_2_, which can facilitate the formation of β-PVDF.

Subsequently, density of states (DOS) analyses were carried out for both the α-phase and β-phase in conjunction with MoS_2_ (Figure 5c,d). It was observed that the DOS overlap between the β-phase and MoS_2_ is more pronounced. Below Fermi level, the β-phase has a stronger peak intensity than that of the α-phase, and the β-phase is closer to the Fermi level. This band feature facilitates more efficient charge transfer between the β-phase and MoS_2_ compared to the α-phase, leading to a stronger binding affinity. This point has been confirmed by the charge transfer between PVDF and MoS_2_, as displayed in Figure 5c,d. These findings substantiate that MoS_2_ promotes the growth of the β-phase.

### 3.2. Piezoelectric Properties of MoS_2_/PVDF Sensor

**Figure 6:**
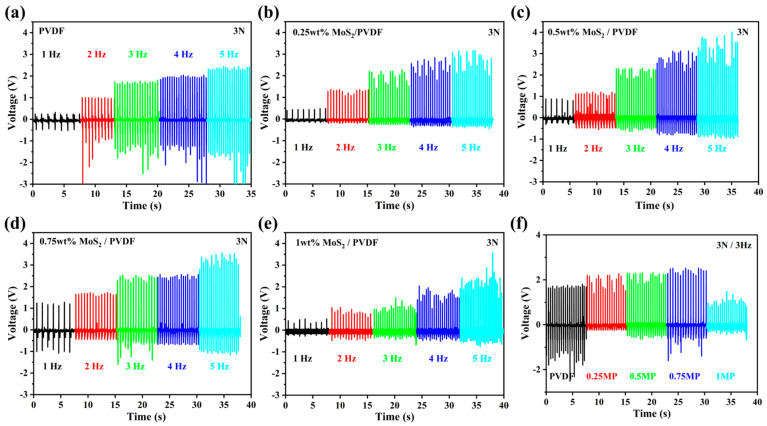
Output voltages of (**a**) PVDF, (**b**–**e**) MoS_2_/PVDF composite sensor at different content of MoS_2_ (0.25, 0.5, 0.75, and 1 wt%), and (**f**) output voltage of PVDF and MP at 3 N and 3 Hz.

The MoS_2_/PVDF composite nanofiber film serves as the piezoelectric layer in the pressure sensor. The schematic diagram of the sensor structure is shown in Figure 1. The electrical signals generated by the MoS_2_/PVDF composite nanofiber film are transmitted through interdigitated electrodes located beneath the film and are subsequently conveyed via conductive wires to the external circuit for signal processing and analysis. By utilizing a mechanically controlled apparatus composed of a cylindrical object with a diameter of 1 cm and a motor, computer-controlled external mechanical force was applied to the sensor, and its electrical signals were recorded using an oscilloscope. To measure the piezoelectric performance of the sensor, several tests were conducted. In the first set of tests, the surface of the sensor was repeatedly struck by the aforementioned mechanical device, maintaining a constant external force of 3 N and observing changes in the sensor’s electrical signals by altering the frequency of strikes. As shown in Figure 6a–e, the output voltages of MoS_2_/PVDF composite sensors with different mass fractions and pure PVDF sensor increased with the rise in frequency. This is because the charge transfer within the sensor remains constant across different vibration frequencies, but the output performance varies due to changes in the speed of the electron flow [31].

For the sake of consolidating the results, the striking frequency was set to 3 Hz, and as evident from Figure 6f, the maximum open-circuit output voltages recorded are 1.8 V for pristine PVDF film and 2.2 V, 2.3 V, 2.5 V, and 1.5 V for MoS_2_/PVDF-based sensors with MoS_2_ loadings of 0.25 wt%, 0.5 wt%, 0.75 wt%, and 1 wt%, respectively. Given the high electrochemical activity of MoS_2_ and its uniform dispersion within the PVDF composite nanofiber membrane, the output voltage was enhanced. When the MoS_2_ content was 0.75 wt%, the MoS_2_/PVDF composite sensor achieved a maximum output voltage of 2.5 V. Increasing the MoS_2_ content beyond this point did not result in an increased output voltage but rather caused a significant decrease. An excess of MoS_2_ in the solution tends to aggregate into small clusters, disrupting the ordered structure and hindering the formation of the β-phase, which impairs electron transport within the composite.

**Figure 7:**
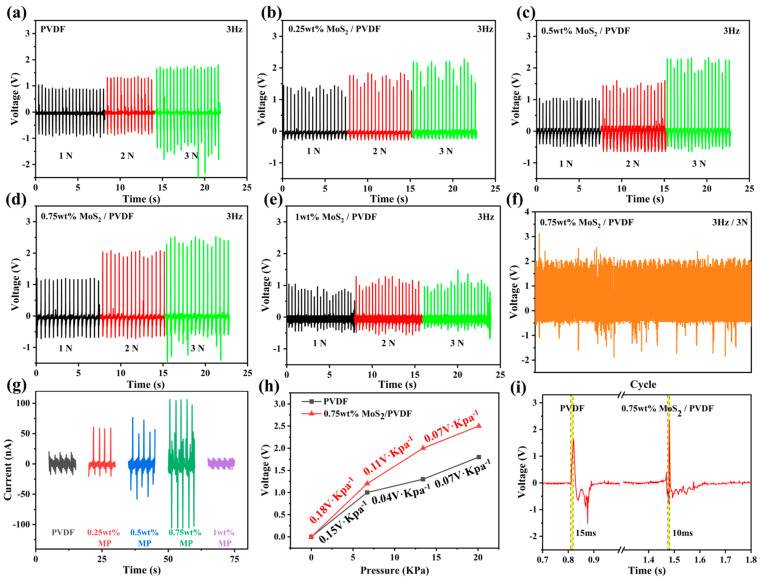
Output voltages of (**a**) PVDF and (**b**–**e**) MP at 3Hz, (**f**) durability testing of MoS_2_/PVDF composite sensor, (**g**) output current of sensors at different concentrations, (**h**) sensitivity, and (**i**) response time of PVDF with 0.75 wt% MP sensor.

To further evaluate the electrical performance of the sensors, compressive forces with varying magnitudes were applied at a constant frequency of 3 Hz, with the corresponding output results presented in Figure 7a–e. The data again indicated that the 0.75 wt% MoS_2_/PVDF composite sensor performed best. Its maximum output voltage under 1 N, 2 N, and 3 N was 1.2 V, 2.0 V, and 2.5 V, respectively (Figure 7d). The output voltage stability of the 0.75 wt% MoS_2_/PVDF composite sensor under periodic impact testing at 3 Hz/3 N demonstrated that there was almost no decline in output voltage after a long duration of 2000 cycles, indicating its practical applicability (Figure 7f). Subsequently, we utilized an electrochemical workstation to measure the output current of the sensor. As shown in Figure 7g, under impact conditions of 3 N force at 3 Hz (with a sensor area of 1.5 cm^2^), the peak currents for PVDF, 0.25 wt% MoS_2_/PVDF (MP), 0.5 wt% MP, 0.75 wt% MP, and 1 wt% MP were 20.2 nA, 60.5 nA, 76.7 nA, 106.0 nA, and 9.4 nA, respectively. These results are consistent with the trends observed in the earlier piezoelectric performance tests. The sensitivity of the sensor is defined as the ratio of the output change (ΔU) to the input change (ΔP) under stable operating conditions, reflecting the measurement accuracy of the sensor. The sensitivity formula is given by [32]:

(2) $$S_{v}=\frac{∆U}{∆P}\;(v\;=\;1,\;2,\;3),$$

where v is a constant. According to this formula, within the pressure range of 0–20 kPa, the voltage sensitivity of the 0.75 wt% MP sensor is higher than that of the pure PVDF sensor, with values of 0.18 V/kPa, 0.11 V/kPa, and 0.07 V/kPa (as shown in Figure 7h). The response time of the sensor is defined as the time it takes for the signal to rise from its initial state to 90% of its peak value [33]. As illustrated in Figure 7i, the response times for the PVDF and 0.75 wt% MP sensors were 15 ms and 10 ms, respectively. The high sensitivity and fast response time of the 0.75 wt% MoS_2_/PVDF composite nanofiber film ensure its effectiveness as a piezoelectric sensor in practical applications. These properties make it particularly suitable for use in various sensing and energy harvesting devices.

**Figure 8:**
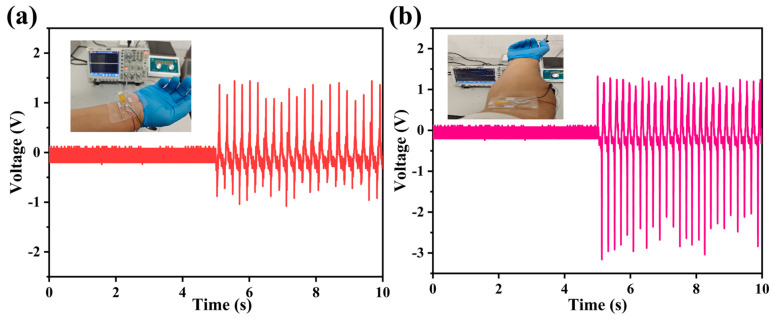
Output voltages of MoS_2_/PVDF composite sensor for (**a**) wrist bending, (**b**) elbow bending.

The practical implementation of MoS_2_/PVDF composite sensors was further investigated through joint motion monitoring. Under static joint conditions (wrist and elbow positions), the sensors generated low-amplitude voltage signals (<0.2 V) with minimal frequency components, as documented in Figure 8a,b. Rapid joint flexion conversely induced substantial electromechanical responses, with output voltage magnitude and frequency increasing proportionally to bending velocity. Maximum performance metrics were recorded at 1.3 V (wrist flexion) and 1.5 V (elbow flexion), demonstrating the sensors’ dynamic responsiveness to biomechanical movements.

With the acceleration of population aging, the prevalence of knee joint issues leading to leg deformities such as genu varum (bow-leggedness) or genu valgum (knock-knees) has become increasingly common among the elderly. These changes in leg morphology significantly affect gait patterns, which in turn alter the foot pressure distribution during ambulation. Compared to younger individuals, older adults exhibit distinct differences in foot pressure distribution while walking. To gain deeper insights into this phenomenon and provide data-driven support for medical and rehabilitation applications, the use of piezoelectric sensors based on MoS_2_/PVDF composite nanofibers for foot pressure monitoring holds significant importance.

**Figure 9:**
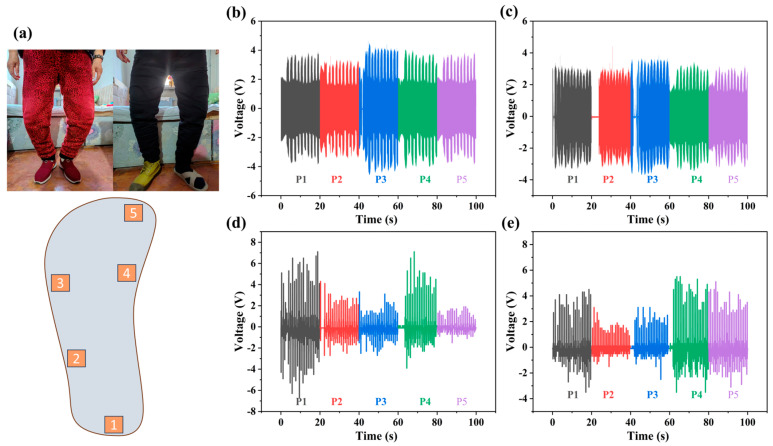
(**a**) Elderly leg types and testing points diagram, testing diagrams for elderly for (**b**) woman and (**c**) man, and (**d**,**e**) testing diagrams for normal young adults.

As illustrated in Figure 9a, we applied the sensors to five key points on the foot (labeled as points 1–5) to monitor the foot pressure distribution during walking for both elderly and young individuals. Through comparative gait analysis, our findings lead us to conclude the following key observations, as detailed in Figure 9b–e. Elderly individuals exhibit a slower walking pace and lower step frequency compared to younger adults. The foot pressure distribution in the elderly is more dispersed, with a particularly prominent pressure peak at point 3. This may be attributed to leg deformities such as genu varum or genu valgum, which cause a shift in the center of gravity and increase the load on the arch region. Compared to younger individuals, the elderly have relatively lower peak pressures, and their pressure distribution is more uniform across the foot. In contrast, young individuals exhibit concentrated foot pressure primarily at point 1 and point 4. This indicates a more natural gait pattern, with pressure distribution that aligns with normal biomechanical principles. Young individuals walk at a faster pace and higher step frequency, resulting in more rapid changes in foot pressure. This study provides foundational data for gait analysis and elderly health management strategies.

## 4. Conclusions

This study utilized the electrospinning method to fabricate MoS_2_/PVDF composite nanofiber films by incorporating varying weight percentages of MoS_2_ into PVDF solutions. It was found that a specific amount of MoS_2_ effectively induced the formation of the β-phase, enhancing the piezoelectric performance of the composite. DFT calculations revealed that the β-phase PVDF has a higher energy, with an energy difference of 0.074 eV/C-atom compared to the α-phase; notably, the 0.75 wt% MoS_2_/PVDF composite nanofiber film exhibited a β-phase content of 70.0%. The resulting sensor generated an output voltage of 2.5 V and an output current of 106.0 nA under an external force of 3 N applied at 3 Hz, demonstrating higher sensitivity compared to pure PVDF sensors and reducing the response time from 15 ms to 10 ms. Additionally, the output voltage remained stable over 2000 cycles, showcasing excellent durability and reliability. Furthermore, the MoS_2_/PVDF composite sensor demonstrated excellent responsiveness in human monitoring applications, indicating its potential for wearable technologies such as smart insoles and health-monitoring devices.

## Data Availability

The original contributions presented in this study are included in the article/Supplementary Materials; further inquiries can be directed to the corresponding author.

## Supplementary Materials

The following supporting information can be downloaded at: https://www.mdpi.com/article/10.3390/s25072085/s1, Figure S1: MoS_2_/PVDF (top) and PVDF (bottom) photos; Figure S2: The fiber size distribution of PVDF (a) and MoS_2_/PVDF (b); Figure S3: Demonstration of the flexibility of piezoelectric sensors; Figure S4: The principle of the interdigitated electrode; Figure S5: (a) testing diagrams for elderly man, (b) testing diagrams for normal young adults.

## References

[1] Tang W., Sun Q., Wang Z.L. (2023). Self-Powered Sensing in Wearable Electronics─A Paradigm Shift Technology. Chem. Rev..

[2] Xiong J., Wang L., Liang F., Li M., Yabuta Y., Iqbal M.A., Mayakrishnan G., Shi J., Kim I.S. (2024). Flexible Piezoelectric Sensor Based on Two-Dimensional Topological Network of PVDF/DA Composite Nanofiber Membrane. Adv. Fiber Mater..

[3] Lee M., Chen C.Y., Wang S., Cha S.N., Park Y.J., Kim J.M., Chou L.J., Wang Z.L. (2012). A Hybrid Piezoelectric Structure for Wearable Nanogenerators. Adv. Mater..

[4] Wei Y., Wu W., Guo R., Yuan D., Das S., Wang Z.L. (2010). Wafer-Scale High-Throughput Ordered Growth of Vertically Aligned ZnO Nanowire Arrays. Nano Lett..

[5] Le A.T., Ahmadipour M., Pung S.-Y. (2020). A review on ZnO-based piezoelectric nanogenerators: Synthesis, characterization techniques, performance enhancement and applications. J. Alloys Compd..

[6] Lim J., Jung H., Baek C., Hwang G.-T., Ryu J., Yoon D., Yoo J., Park K.-I., Kim J.H. (2017). All-inkjet-printed flexible piezoelectric generator made of solvent evaporation assisted BaTiO3 hybrid material. Nano Energy.

[7] Fu D., Sogen S., Suzuki H. (2024). Intrinsic Piezoelectricity of PZT. ACS Appl. Electron. Mater..

[8] Chen X., Han X., Shen Q.-D. (2017). PVDF-Based Ferroelectric Polymers in Modern Flexible Electronics. Adv. Electron. Mater..

[9] Shepelin N.A., Glushenkov A.M., Lussini V.C., Fox P.J., Dicinoski G.W., Shapter J.G., Ellis A.V. (2019). New developments in composites, copolymer technologies and processing techniques for flexible fluoropolymer piezoelectric generators for efficient energy harvesting. Energy Environ. Sci..

[10] Zheng Z., Wang X., Hang G., Duan J., Zhang J., Zhang W., Liu Z. (2024). Recent progress on flexible poly(vinylidene fluoride)-based piezoelectric nanogenerators for energy harvesting and self-powered electronic applications. Renew. Sustain. Energy Rev..

[11] Bairagi S., Ali S.W. (2020). Investigating the role of carbon nanotubes (CNTs) in the piezoelectric performance of a PVDF/KNN-based electrospun nanogenerator. Soft Matter.

[12] Yang X., Wang Y., Qing X. (2019). A flexible capacitive sensor based on the electrospun PVDF nanofiber membrane with carbon nanotubes. Sens. Actuators A Phys..

[13] Gu J., Lee D., Oh J., Si H., Kim K. (2024). Synergy of Polydopamine-Assisted Additive Modification and Hierarchical-Morphology Poly(Vinylidene Fluoride) Nanofiber Mat for Ferroelectric-Assisted Triboelectric Nanogenerator. Adv. Fiber Mater..

[14] Jiang J., Wan L., Li L., Li P. (2024). High-Performance Piezoelectric Nanogenerator of BTO-PVDF Nanofibers for Wearable Sensing. Macromol. Rapid Commun..

[15] Du X., Zhou Z., Zhang Z., Yao L., Zhang Q., Yang H. (2022). Porous, multi-layered piezoelectric composites based on highly oriented PZT/PVDF electrospinning fibers for high-performance piezoelectric nanogenerators. J. Adv. Ceram..

[16] Liang M., Wang J., Su L., Xin X., Chen Z., Zhang Y., Jiao Y., Luan X., Chen L., Yao Z. (2024). Versatile Lamellar Wrap-Structured PVDF/PZT/CNTs Piezoelectric Sensor for Road Traffic Information Sensing, Monitoring, and Energy Harvesting. Chem. Eng. J..

[17] Kim M., Fan J. (2021). Piezoelectric Properties of Three Types of PVDF and ZnO Nanofibrous Composites. Adv. Fiber Mater..

[18] Zhang J., Yang T., Tian G., Lan B., Deng W., Tang L., Ao Y., Sun Y., Zeng W., Ren X. (2024). Spatially Confined MXene/PVDF Nanofiber Piezoelectric Electronics. Adv. Fiber Mater..

[19] Wang S., Yu Z., Wang L., Wang Y., Yu D., Wu M. (2023). A core-shell structured barium titanate nanoparticles for the enhanced piezoelectric performance of wearable nanogenerator. Appl. Energy.

[20] Zhang Y., Zhang R., Guo Y., Li Y., Li K. (2024). A review on MoS_2_ structure, preparation, energy storage applications and challenges. J. Alloys Compd..

[21] Kresse G., Joubert D. (1999). From ultrasoft pseudopotentials to the projector augmented-wave method. Phys. Rev. B.

[22] Kresse G., Hafner J. (1993). Ab initio molecular dynamics for liquid metals. Phys. Rev. B.

[23] Perdew J.P., Burke K., Ernzerhof M. (1996). Generalized Gradient Approximation Made Simple. Phys. Rev. Lett..

[24] Blöchl P.E. (1994). Projector augmented-wave method. Phys. Rev. B.

[25] Martins P., Lopes A.C., Lanceros-Mendez S. (2014). Electroactive phases of poly(vinylidene fluoride): Determination, processing and applications. Prog. Polym. Sci..

[26] Singh H.H., Singh S., Khare N. (2017). Design of flexible PVDF/NaNbO3/RGO nanogenerator and understanding the role of nanofillers in the output voltage signal. Compos. Sci. Technol..

[27] Yang Y., Pan H., Xie G., Jiang Y., Chen C., Su Y., Wang Y., Tai H. (2020). Flexible piezoelectric pressure sensor based on polydopamine-modified BaTiO3/PVDF composite film for human motion monitoring. Sens. Actuators A Phys..

[28] Kim S.-H., Ha J.-W., Lee S.G., Sohn E.-H., Park I.J., Kang H.S., Yi G.-R. (2019). Fluorinated Titania Nanoparticle-Induced Piezoelectric Phase Transition of Poly(vinylidene fluoride). Langmuir.

[29] Singh D., Choudhary A., Garg A. (2018). Flexible and Robust Piezoelectric Polymer Nanocomposites Based Energy Harvesters. ACS Appl. Mater. Interfaces.

[30] Xue X., Deng P., Yuan S., Nie Y., He B., Xing L., Zhang Y. (2013). CuO/PVDF nanocomposite anode for a piezo-driven self-charging lithium battery. Energy Environ. Sci..

[31] Shi S., Pan Z., Cheng Y., Zhai Y., Zhang Y., Ding X., Liu J., Zhai J., Xu J. (2022). Three-dimensional polypyrrole induced high-performance flexible piezoelectric nanogenerators for mechanical energy harvesting. Compos. Sci. Technol..

[32] Fan W., Lei R., Dou H., Wu Z., Lu L., Wang S., Liu X., Chen W., Rezakazemi M., Aminabhavi T.M. (2024). Sweat permeable and ultrahigh strength 3D PVDF piezoelectric nanoyarn fabric strain sensor. Nat. Commun..

[33] Mokhtari F., Cheng Z., Raad R., Xi J., Foroughi J. (2020). Piezofibers to smart textiles: A review on recent advances and future outlook for wearable technology. J. Mater. Chem. A.

