# Supplementary material for: Exploring the Synergistic Effects of MoS2 and PVDF for Advanced Piezoelectric Sensors: A First-Principles Approach
Source: Sensors (Basel). 2025 Mar 26;25(7):2085. doi: 10.3390/s25072085 (PMC11991512; doi:10.3390/s25072085)
Supplement: Supplementary file 1 [file sensors-25-02085-s001.zip › sensors-3519094-supplementary.pdf]

Exploring the Synergistic Effects of MoS_2 and PVDF for Advanced Piezoelectric Sensors: A First-Principles Approach

Rui Li[1,2,*], Juqi Wang[1,2], Aolin Li[1], Quanbin Ma[1,2], Shi Feng[1,2],Bo Ran[1,2], Lingling Zhang[3,*]

[1] Xinjiang Key Laboratory of Solid-State Physics and Devices, School of Physics Science and Technology, Xinjiang University, Urumqi 830046, China;
[2] School of Material Science and Engineering，Xinjiang University, Urumqi 830046, China;
[3] Center for Evidence-Based and Translational Medicine, Zhongnan Hospital of Wuhan University, Wuhan 430071, China;
* Correspondence: lirui@xju.edu.cn (Rui Li); llzhang@whu.edu.cn (Lingling Zhang)

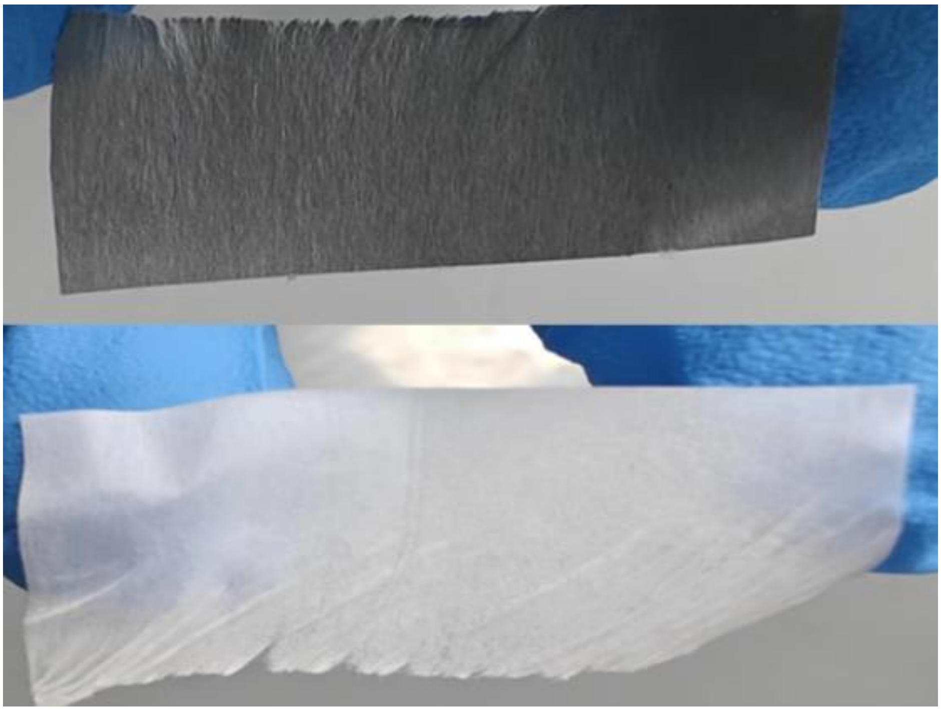

Figure S1: MoS_2/PVDF (top) and PVDF (bottom) photos

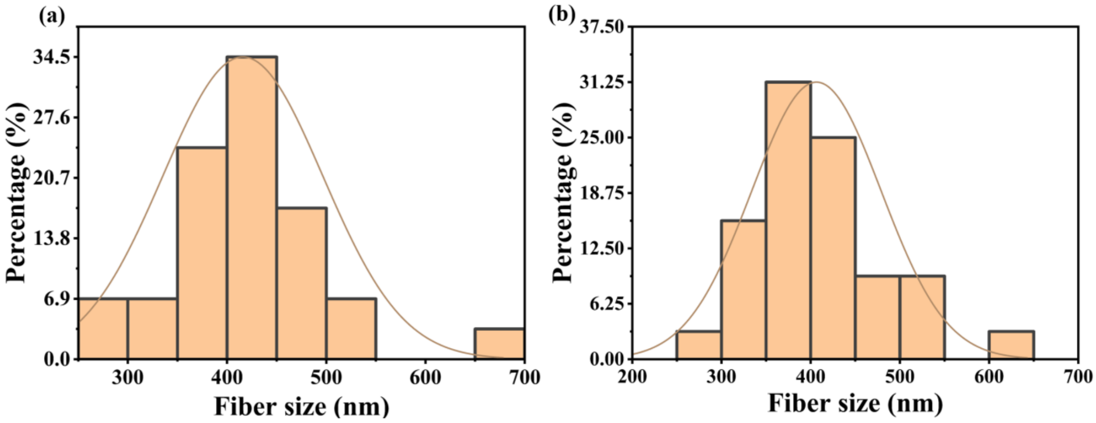

Figure S2: The fiber size distribution of PVDF (a) and MoS_2-PVDF (b).

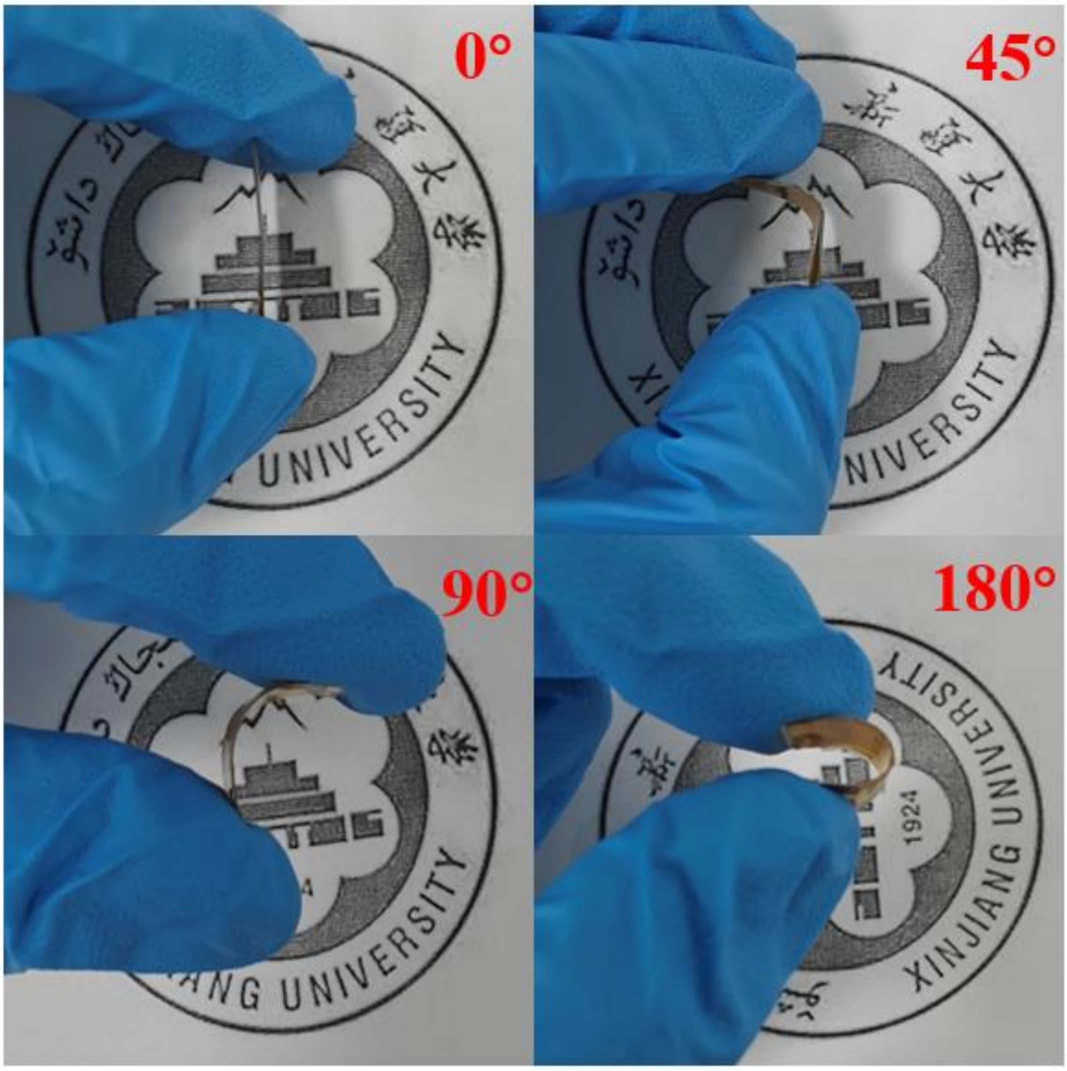

Figure S3: Demonstration of the flexibility of piezoelectric sensors

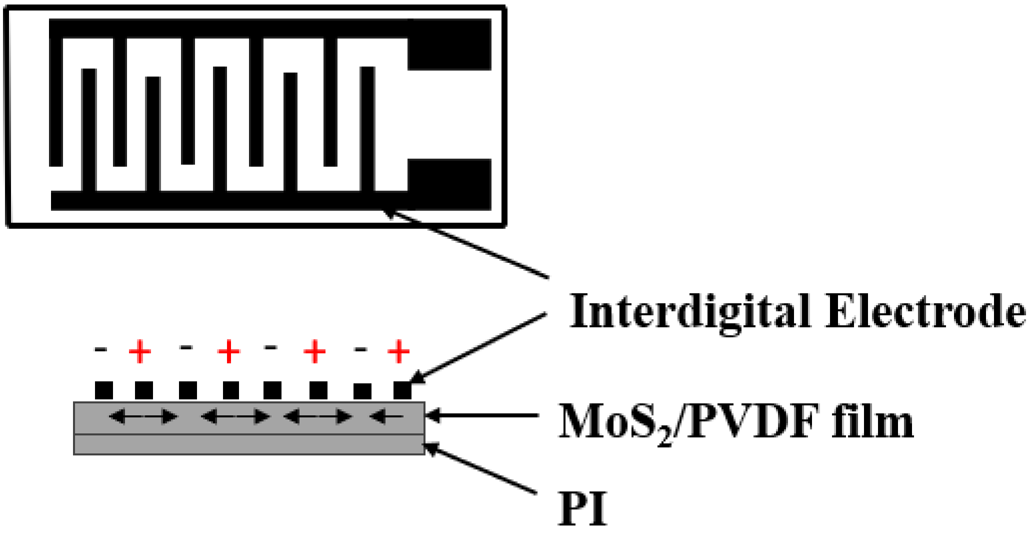

Figure S4: The principle of the interdigitated electrode

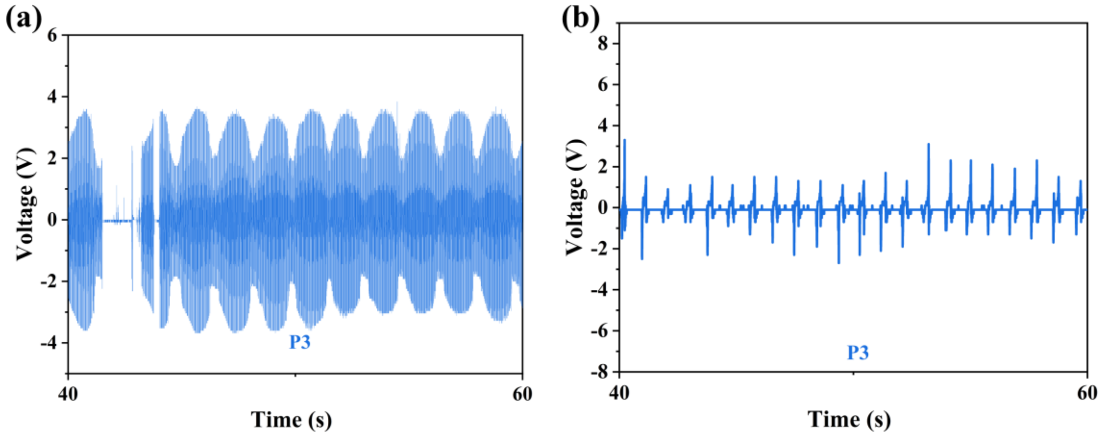

Figure S5. (a)testing diagrams for elderly man,(b) testing diagrams for normal young adults.
